# Executive functions in preschool children with moderate hyperphenylalaninemia and phenylketonuria: a prospective study

**DOI:** 10.1186/s13023-023-02764-9

**Published:** 2023-07-03

**Authors:** Laetitia Paermentier, Aline Cano, Brigitte Chabrol, Arnaud Roy

**Affiliations:** 1 Reference Center of Hereditary Metabolic Diseases, AP-HM, La Timone University Hospital, 264 rue Saint Pierre, 13005 Marseille, France; 2 Department of Paediatric Neurometabolism, AP-HM, La Timone University Hospital, 264 rue Saint Pierre, 13005 Marseille, France; 3 Pays de la Loire Psychology Laboratory, EA4638, Faculty of Arts, Languages and Humanities, University of Angers, 49000 Angers, France; 4 Learning Disabilities Referral Center and Nantes Neurofibromatosis Competence Center, for Women, Children and Adolescents, Nantes University Hospital, 44000 Nantes, France

**Keywords:** Hyperphenylalaninemia, Phenylketonuria, Moderate hyperphenylalaninemia, Preschool children, Executive function, BRIEF-P

## Abstract

**Background:**

The risk of neuropsychological disorders appears to be high in hyperphenylalaninemia (HPA). The hypothesis of executive function impairment is prominent in accounting for the neuropsychological phenotype in phenylketonuria (PKU) and is suspected in moderate hyperphenylalaninemia (MHP). However, the issue of early onset of executive disorders remains. The aim of this study was to explore the hypothesis of early executive dysfunction in HPA patients and the possible links with certain metabolic variables according to the new international classifications for patients with PKU and MHP. A group of 23 HPA children (12 PKU, 11 MHP) aged 3 to 5 years was included and compared to 50 control children. The two groups were comparable in terms of socio-demographics (age, sex, parental education level). Executive functions were assessed using performance-based tests and daily life questionnaires (parents and teachers).

**Results:**

Preschool HPA patients have comparable executive scores to control subjects. In contrast, PKU patients score significantly worse than MHP patients on 3 executive tests (verbal working memory, visual working memory and cognitive inhibition. There is no executive complaints in daily life (parents and teachers) for the 2 groups of patients. In addition, 3 correlations were identified between executive scores and Phe levels at inclusion, mean Phe level and variability of Phe levels throughout life.

**Conclusions:**

Thus, there appears to be evidence of early executive dysfunction in PKU preschool-children, but not in MHP children. Occasionally, certain metabolic indicators can predict executive difficulties in young children with PKU.

## Background

Phenylketonuria (PKU) is an autosomal recessive inherited metabolic disorder with a prevalence of 1 in 10,000 births in Europe caused by a deficiency of phenylalanine hydroxylase (PAH). Management recommendations have recently been drafted at the American [1] and European [2] levels. A distinction is now made between patients with PKU (phenylalanine (Phe) levels ≥ 360 μmol/L) and with moderate hyperphenylalaninemia (MHP) (120 μmol/L > Phe < 360 μmol/L). Neonatal screening for PKU makes it possible to rapidly implement specific management to reduce plasma Phe levels. For MHP patients, a residual amount of enzymatic activity would be present thereby avoiding severe neurodevelopmental disorders which can occur if PKU is left untreated [3].

Neonatal treatment can prevent the development of encephalopathy related to hyperphenylalaninemia (HPA). However, recent studies have shown a «suboptimal» cognitive outcome. The intelligence quotient (IQ) in the PKU is average but significantly lower than in the control population e.g., [4]. Additionally, processing speed, fine motor skills, and episodic memory impairment are observed in patients with PKU [5]. In subjects with MHP, these cognitive functions are likely to be relatively preserved e.g., [6, 7].

Impairment in executive functions (EFs) may explain the neuropsychological phenotype observed in PKU e.g., [8] and is suspected in MHP [9, 10].

EFs refer to a variety of high-level functions required to perform goal-oriented behaviours, primarily involving the prefrontal cortex and its networks. They allow the individual to adapt to new situations, particularly when automatisms are insufficient to carry out the task at hand [11].

However, very little research has examined the incidence of executive dysfunction during preschool years, with mixed results (see Table 1). In all studies involving preschool-age PKU patients, performance was below that of the control group or the test norm [12, 13]. There are also more complaints in the questionnaires about flexibility and control [14]. In MHP patients, the results are contradictory: one study found no disorders [15], while the other reported a continuum of impairment between PKU and MHP patients compared to controls [16]. Limitations inherent to the studies include the fact that the classification of PKU/MHP was variable with Phe levels ranging between 240 and 600 µmol/l for MHP [16]. Furthermore, the assessment of EFs is often limited [17] and only one study has combined performance tests with parental daily life measures [14].

**Table 1:** Studies on executive functions with preschool-aged children with HPA

Study	Patients Age	Matched control group ^a,b,c,d,e^	Tasks	Variables studied	Conclusions
Welsh et al. [13]	PKU: 11 Mean age: 4.64	C ^a,b,c,d,e^	FLV, TOH, VST, MPT	Phe(T) Phe (V)	PKU < C for EF correlated with Phe (T) et Phe (V)
Diamond et al. [16]	PKU = 37 MHP (240–600 μmol/l) = 25 longitudinal study: 6 months to 7 years	C ^a, b, c^ general population siblings	DNMT, 3P, LBT, CORSI, AB, OR, 3-6B, DN, TT	Phe(V), Phe (1), Tyr (V), Phe/Tyr (V)	EF: PKU < MHP < C
Arnold et al. [12]	PKU = 18 Mean Age: 47 months (12–101)	N	AB, OR, VST, FLV, FS, B, M	Phe(T), Phe(V), Phe(S)	EF: 1SD below norms Correlated with Phe (S)
Anderson et al. [14]	PKU = 34 Age: 5–18 years	C ^a, c^	BRIEF, TOL, CNT, COWAT, RCFT		BRIEF: PKU > C in shift and monitoring scale Flexibility: PKU < C
Cappelleti et al. [17]	PKU = 35 Mean age: 11.5 (6.2)	N	TOL, CBCL	Phe (T), Phe/Tyr (T)	Deficit in planification < 10 years Correlated with Phe (T)
Sharman et al. [38]	MHP = 4 (320–550 μmol/l) Mean age: eval 1: 4.66; eval 2: 12.45	N	Eval 1: RCFT, WISC- DS, NEPSY-2 CI Eval 2: RCFT, WISC- DS, NEPSY-2 CI NEPSY-2 INH, BRIEF parents		MHP = N

Patients: *MHP* Patients with moderate hyperphenylalaninemia, *PKU* Patients with classical phenylketonuria

Matched control group: a: age, b: gender, c: sociocultural level, d: IQ, e: low level process, C: control subjects, *N* Testing norm

Tasks: 3-6B: Three or Six Boxes Task, 3P: Three Pegs Task, AB: AB Task, B: Blocks, *BRIEF* Behavior Rating Inventory of Executive Function, *CBCL* Child Behavior Checklist, *CNT* Contingency Naming Test, *COWAT* Controlled Oral Word Association Test, *DN* Day-Night Stroop-Like Test, *DNMT* Delayed Non Matching to sample test, *FLV* Verbal fluency task, *FS* Finger Sequencing, *LBT* Line Bisection Task, *M* Mazes, *MPT* Motor planning task, *NEPSY* Bilan neuropsychologique de l'enfant, *NEPSY-2 Inh* Inhibition Nepsy 2, *NEPSY-2 CI* Compréhension de Consignes Nepsy 2, *OR* Object Retrieval Task, *RCFT* Rey-Osterrieth Complex Figure Test Copy, *TOH* Tour of Hanoi, *TOL* Tower of London, *VABS* Vineland Adaptative Behavior Scales, *VST* Visual search test, *WISC-DS* Digit Span Wisc

Variables studied: *Phe* phenylalanine rate, *Phe/Tyr* Phenylalanine/Tyrosine ratio, *Tyr* Tyrosine rate, (1) average rate first month of life, (B): birth rate, (S): variability of Phe during lifetime, (T): rate at evaluation, (V): average rate from birth to evaluation

Conclusions: *EF* Executive functions, *SD* Standard deviation. In the conclusion section: for performance-based tests: if the results are lower than the norm or the control subjects, the sign " < " is used and vice versa. For the daily life questionnaires: if there are more parental complaints than the norm, we use the sign " > " and vice versa

Several studies have investigated the relationship between biochemical markers and cognitive impairment in early-treated PKU. Currently, it appears that the best predictor of neuropsychological disorders, and especially executive disorders, is the mean / median levels of Phe during lifetime and the variability of these levels over time. Recent studies have also shown a possible link between EFs disorders and an elevated Phe/Tyrosine (Tyr) ratio over time or at the assessment time [see 5].

In this study, we propose to explore the hypothesis of early executive dysfunction in HPA patients, while distinguishing PKU and MHP patients according to the new classifications, and to examine the links between EFs and certain metabolic variables (mean Phe levels and Phe/Tyrosine ratio over the course of a lifetime, variability of Phe levels over the course of a lifetime, Phe levels and Phe/Tyrosine ratio at inclusion). We used a protocol that combined several executive performance-based tasks with daily life questionnaire (parents and teachers). It is crucial to identify EFs disturbances from preschool age to promote their management given their profound impact on environmental adaptation, academic success and, learning [18].

Based on previous studies, we hypothesized that children in the HPA group would manifest impaired EFs (and increased reports in daily life) compared to the control group (1). We also expected to observe a continuum of executive impairment with increasing severity of HPA, resulting in poorer test performance and increased reports on daily life questionnaires in PKU patients compared to MHP patients (2). Lastly, associations are expected between metabolic indicators and test/questionnaire scores: the higher the levels, the worse the executive performance/the higher the complaints (3).

## Methods

### Participants

Children with PKU and MHP were recruited from the Referral Center for Hereditary Metabolic Diseases in the Marseille University Hospital in France. Patients were seen between February 2017 and December 2018 during routine follow-up consultations for their pathology. The study was approved by the South Mediterranean Committee for the Protection of Individuals (RO-2016/16) and registered with the French Data Protection Authority (CNIL; 2,024,752). Prior to their participation, parents and children were given oral and written information and signed a consent form.

To be included, children had to be between 3 and 5 years 11 months of age and monitored for PKU or MHP detected in the neonatal period by new-born screening. The patients were classified between PKU and MHP based on confirmatory Phe level after a positive screening on Guthrie card at D3 (> 360 μmol/l).The exclusion criteria for the study were as follows: late diagnosis or massive non-adherence to treatment for the PKU (1), other neurological or psychiatric disorders (2), insufficient level of French proficiency (3), sensory disorders preventing assessment (4), medication or substance abuse incompatible with assessment (5) absence of parental consent signature (6), neuropsychological evaluation performed less than 6 months prior (7). Twenty-five families were asked to participate in this study, one refused to sign the consent form, and one child (very preterm) was excluded. No children were excluded for massive non-adherence to the regimen in PKU (rates outside the recommendations on a very regular basis and no follow-up of Phe rates on guthrie cardboard). The final patient sample consisted of 12 PKU children (10 girls, 2 boys; Mean (M): 4.6 years, Standard Deviation (SD): 10 months) and 11 MHP children (6 girls, 5 boys; M: 4.3 years, SD 10.5 months).

The healthy control sample consisted of 55 volunteer children recruited from nursery schools and aged between 3 and 5 years 11 months. The exclusion criteria were the same as for PKU and MHP children, in the absence of PKU/MHP naturally. Five control children were excluded to match the sex, age, and parental education level between the control and patient groups. Therefore, the final control group consisted of 50 preschool-age children. All the families approached agreed to participate in the study.

## Materials

### Executive tests

A set of 7 experimental performance-based tasks was used to assess EFs combined with a daily life questionnaire. Several classic tests normally considered to assess the main components of EFs (inhibition, working memory and flexibility) were used [11, 18, 19].

*The Sun-Moon Stroop *[20] assesses verbal inhibition*.* This test consists of two pages, each with 6 lines and 5 items per line representing suns and moons alternating in a random fashion. In the control phase, the child is asked to name the pictures shown, sun for sun and moon for moon, as quickly as possible over a period of 45 s. If the child makes a mistake, the child is asked to correct himself or herself before proceeding. In the inhibition phase, the child must prevent the predominant response and say "sun" when they see a "moon" or vice versa as quickly as possible for 45 s. It is not corrected in case of error. An interference score is calculated ((number of items in interference condition – number of items in control condition)/number of items in control condition) and an error score (number of errors in interference condition – number of errors in control condition). The higher the interference score, the better the inhibition process. On the contrary, the higher the error score, the more it suggests executive difficulties.

Motor inhibition was measured via an adaptation of the *Hand-Game *[21]. The control condition is composed of 6 randomly presented items where the child is asked to reproduce the same gesture as the examiner: if the examiner shows his/her fist, and the child shows his/her fist, if the examiner shows a pointing finger, the child shows a pointing finger. The interfering condition is composed of 15 randomly presented items where the child must perform the opposite gesture of the examiner: point a finger if the child sees a fist and vice versa. Feedback is given for each trial: “good, go on” or in the event of a mistake, a reminder of the instructions. One point is awarded for each correct answer. The number of errors and self-corrections is recorded. The number of items in the task and the scoring conditions differ from the original procedure: the 15 items are proposed systematically in contrast to the original task where the stopping criterion was to have six correct answers in a row over a maximum of 15 trials [21].

To assess auditory-verbal working memory, the *Digit Span* test was administered [22]. First, the child must repeat a series of numbers in the same order as the examiner (forward span). Then the child repeats the series of numbers in reverse (backward span). The longest series of digits correctly recalled twice in each condition is recorded.

Visual working memory was measured using the *spatial Span* test [23]. On a board with 10 randomly placed cubes, the child points to a series of cubes in an order previously set in a similar way to the examiner (forward spatial span) and then in the opposite order to that presented by the examiner (backward spatial span). The longest series passed twice is encoded for both parts of the test.

The *Dimensional Change Card Sort* (DCCS) test [24] is a card sorting test that assesses cognitive flexibility. Two target cards are presented to the child: a blue rabbit and a red boat. Then the child sorts 6 cards according to a previously chosen criterion (colour or shape) and counterbalanced between the groups of children. If the child scores ≥ 5 in this pre-test phase, the child will then have to sort 6 cards again according to the other criterion. If the child passes this post-test phase (score ≥ 5/6) the child will complete the "border" section where the sorting criterion will change according to whether the card has a black border or not (black border = colour, no border = shape). Twelve cards are offered in this section, considered successful if the child scores ≥ 9/12. The overall score for this test is a maximum of 3 points, 1 point per section.

The *Brixton* test for preschoolers [25] investigates flexibility and rule deduction skills. An adaptation of this test is proposed here: thirty clay pots are placed in a line, and the child is required to determine which pot is hiding a mouse. It is placed under the first pot of the row (either on the right or on the left) at the beginning of the task. The examiner reminds the child of the previous location on each trial to limit the burden on the working memory. After each attempt, the correct location is shown. The change in movement rule is made three times (+ 1, − 2, + 3) and is counterbalanced between the groups. Eight movements are made per rule. The overall score corresponds to the number of correct moves for the 3 movement rules (*n* = 24).

Finally, affective decision making was assessed via the *Children's Gambling Task* (CGT) [26]. The purpose of this test is to win as many sweets as possible (Skittles®, compatible with the patient’s diet, are used to represent wins and losses). Two sets of cards (lines or dots) are available, each containing two packs of 50 cards: 1 advantageous (constant gain of 1, loss of 0 or 1) and 1 disadvantageous (constant gain of 2, loss of 0 to 6). Each set and each package (advantage to the left or right) are counterbalanced between trials, allowing for 4 different arrangements. Fifty trials are performed after 6 example items. At each trial, the child is asked to choose a card from one of the piles. An overall proportion score is calculated [(number of advantageous choices/50) – (number of disadvantageous choices/50)]. The higher the score, the more advantageous choices the child has made and therefore the greater the perceived gain.

The BRIEF-P questionnaire, which assesses EFs in daily life, was also completed for the parent and teacher versions [27]. It contains 63 questions which are grouped into five scales: Inhibition, Flexibility, Emotional Control, Working Memory, Planning / Organization. These scales are grouped into three generic indices: Inhibitory Control Index (ICI), Flexibility Index (FI), and Emergent Metacognition Index (EMI). Finally, a Global Executive Composite (GEC) score summarises these scales and indices. They were calculated according to French standards (*T* score mean = 50, standard deviation = 10). The higher the score, the more it symbolises an executive impairment (significance *T* score ≥ 65). It should be noted that in this study, only the scores on the five clinical scales and the GEC were considered. The negative and inconsistency scores were analysed according to the recommendations of the manual to ensure the validity of the questionnaires.

### Metabolic variables

Patients followed for HPA have regular monitoring of their capillary phenylalanine level, on samples taken on a guthrie card at home and sent to the laboratory of the monitoring centre. The samples are taken once a week for PKU children and once a week to once a month for MHP children. Plasma levels of phenylalanine and tyrosine (and the entire aminoacidogram) are assessed once a year by plasma amino acid chromatography, carried out during the annual biological nutritional check-up. The different metabolic data that interested us (Phe and Tyr levels) were collected retrospectively from the neonatal period to the study inclusion. Mean Phe and mean Phe/Tyr ratio were the mean of all available Phe levels and Phe/Tyr ratio for each child (including the neonatal rates for the mean Phe). The number of blood Phe levels available across the lifetime ranged from 47 to 278 (M = 96.7, SD = 70.9), with total 2321 Phe levels. The number of available Phe/Tyr ratio ranges from 1 to 9 per patient (M = 5.3, SD = 1.6), with total 126 Phe/Tyr ratio. To assess the variability of Phe, we used the Standard Deviation of Phe (SD Phe) which indicated the degree of dispersion in Phe around the mean.

### Procedure

The PKU and MHP patients were received twice to perform the full neuropsychological assessment (2 × 2 h) in a quiet room. The first appointment took place during their scheduled annual visit to the Referral Centre for Hereditary Metabolic Diseases, during which a blood test is systematically carried out to assess nutritional and metabolic status. Metabolic parameters (Phe and Phe/Tyr levels at the time of inclusion) were collected following this assessment. The second appointment was scheduled within 6 months during a medical visit for PKU patients, or only to continue with the neuropsychological evaluation for MHP patients. The assessment of the control subjects took place in their homes. The tests were administered in the same order for each child: intellectual efficiency and then executive tests (Sun-Moon Stroop, Spatial Span, DCCS, Hand Game, Digital Span, Preschool Brixton, CGT). The BRIEF-P questionnaires were given out during the first appointment in duplicate: a parent version and a teacher version. These were collected at the second appointment.

### Statistical analyses

Statistical analyses were performed using JASP software (0.16.0). Non-parametric tests were chosen due to the small numbers.

The Mann–Whitney test for independent samples was used to compare test scores and questionnaire between the HPA and control groups and between the PKU and MHP subgroups. Effect sizes were calculated using biserial correlation coefficients. These coefficients were interpreted according to the recommendations of Cohen [28]: small if 0.2 ≤ *d* < 0.5; moderate if 0.5 ≤ *d* < 0.8; large if *d* ≥ 0.8. In addition, non-parametric Spearman's correlations (*r*) were performed to investigate the relationships between the performance tests and questionnaire results of HPA, PKU and MHP patients with selected metabolic variables (mean lifetime Phe levels, mean lifetime Phe/Tyr ratio, Phe levels at inclusion and variability of Phe levels over time). To limit type 1 measurement errors due to multiple comparisons and correlations, a score of *p* < 0.01 was considered significant, a score of *p* < 0.05 as a trend.

## Results

### HPA groups

#### Sample characteristics

The demographic and clinical characteristics of HPA and control patients are summarized in Table 2. The two groups are comparable in terms of age, sex (distribution of girls and boys) and parental education level (mean years of parental education since starting primary school). An IQ assessment was performed using the Wechsler Preschool and Primary Scale of Intelligence, Fourth Edition (WPPSI-IV [29]). French norms were used to calculate standard scores (*M* = 100; *SD* = 15). The control children completed only two subtests: Cubes and Information (*M* = 10, *SD* = 3). Means for all WPPSI-IV indices (Verbal Comprehension Index (VCI), Visual Spatial Index (VSI), Fluid Reasoning Index (FRI), Working Memory Index (WMI), Processing Speed Index (PSI)) and the Full Scale Intelligence Quotient (FSIQ) were within the expected range for the age (score between 90 and 109) in the HPA group. There were no significant differences between the two groups (HPA vs controls) in scores on the WPPSI-IV Cubes and Information test.

**Table 2:** Demographic and clinical data of the HPA and healthy control groups

	HPA (*n* = 23)			*n*	C (*n* = 50)			Mann–Whitney		Effet size
	M	SD	Range		M	SD	Range	U	*p*	
Age (months)	53.2	10.3	36–71	23	55.7	10.6	36–71	650.500	0.373	
Mean PEL (years)	12.9	2.4	8.5–18.5	23	13.6	2.1	11–20	686.000	0.187	
Girls/Boys	16/7			23	25/25			Chi^2^ = 2.449	0.118	
Phe D3 (μmol/l)	391	243	156–960	19						
Phe D10 (μmol/l)	734	713	186–2600	23						
Inclusion Phe (μmol/l)	368	225	138–984	23						
Inclusion Phe/Tyr Ratio	8	9	1–39	23						
Mean Phe (μmol/l)	300	94	173–618	23						
Mean Phe/Tyr Ratio	10	10	2–41	23						
Variability Phe (μmol/l)	140	104	28- 393	23						
Blocks (WPPSI-IV)	9.6	2.2	3–13	23	11.1	2.6	7–19	727.000	0.068	0.264
Information (WPPSI-IV)	10.3	2.2	6–14	23	10.8	2.3	4–16	634.500	0.478	0.103
VCI	106	12.8	87–129	23						
VSI	97.5	10.9	72–112	22						
FRI	95.5	8.7	80–111	14						
WMI	96.3	9.8	75–109	21						
PSI	94.8	10.6	77–118	12						
FSIQ	98.8	11.7	77–118	23						

*C* Controls, *FRI* Fluid reasoning index, *FSIQ* Full-scale intelligence quotient, *HPA* Children with hyperphenylalaninemia, *Inclusion Phe* Phe level at the study inclusion, Inclusion Phe/Tyr ratio ratio Phe/Tyr at the study inclusion, *M* Mean, Mean Phe: mean Phe level during lifespan; Mean Phe/Tyr ratio: mean Phe/Tyr level during lifespan; n: headcount; *PEL* Parental education level, *Phe D3* Phe level at day 3 of live, *Phe D10* Phe level at day 10 of live, *PSI* Processing speed index, *SD* Standard deviation, *Variability Phe* Phe level variability during lifespan, *VCI* Verbal comprehension index, *VSI* Visual spatial index, *WMI* Working memory index

### Executive functions

In Table 3, the results of the EF assessment tests and the BRIEF-P questionnaires (parents and teachers) for HPA patients and controls are presented. HPA patients had a higher error score (*p* < 0.05, trend) and a lower self-correction score (*p* < 0.01) in the Hand Game. On the contrary, they had a lower error score than controls in the Sun-Moon Stroop (*p* < 0.01), a higher overall success score on the DCCS (*p* < 0.01) and a higher overall proportion score on the CGT (*p* < 0.05, trend). The two groups had comparable scores on the Sun-Moon Stroop interference score, the forward and backward digit span, the forward and backward visuospatial span, and the overall score on the Brixton Preschool. The effect sizes are small overall. Regarding the BRIEF-P parent questionnaires, there were no significant differences between the two groups (HPA vs controls). Regarding teachers, the inhibition scale score tended to be higher in patients with HPA compared to controls (*p* = 0.015, small effect size).

**Table 3:** Score on executive performance-based tests and BRIEF-P ratings in preschool children with HPA and healthy controls

	HPA			C			Mann–Whitney		Effet size
	M	SD	*n*	M	SD	N	U	*p*	r
*Sun-moon stroop*									
Interference score	− 0.398	0.224	20	− 0.460	0.204	50	402.000	0.205	− 0.196
Error score	1.300	7.057	20	5.120	7.311	50	704.500	0.008*	0.409
*Hand-game*									
Uncorrected errors	2.286	3.133	21	1.042	2.617	48	341.000	0.019(*)	− 0.323
Self-corrected errors	0.762	1.480	21	2.250	2.139	48	732.000	0.002*	0.452
*Digit span*									
Forward span	3.286	0.956	21	3.260	0.803	50	476.000	0.498	− 0.093
Backward span	1.048	1.161	21	1.429	1.258	49	592.500	0.277	0.152
*Spatial span*									
Forward span									
Backward span	1.364	1.364	22	1.796	1.354	49	629.000	0.241	0.168
*DCCS*	3.318	0.945	22	3.265	1.132	49	529.500	0.906	− 0.018
Success score	2.227	0.528	22	1.720	0.640	50	340.500	0.002*	− 0.381
*Brixton preschool*									
Total success score	12.864	6.628	22	13.620	5.931	50	611.500	0.455	0.112
*CGT*									
Overall proportion score	0.244	0.413	22	0.029	0.450	50	389.000	0.049(*)	− 0.293
*BRIEF-P (parents)*									
Inhibit	51.167	15.565	18	45.625	8.584	48	357.500	0.286	− 0.172
Emotional control	52.722	12.111	18	47.875	10.111	48	325.500	0.125	− 0.247
Shift	54.389	14.955	18	48.896	9.887	48	337.000	0.171	− 0.220
Working memory	52.667	13.827	18	46.646	8.360	48	325.000	0.124	− 0.248
Planning/organize	53.278	14.720	18	47.375	9.843	48	335.000	0.164	− 0.225
GEC	53.056	15.318	18	46.063	8.811	48	315.500	0.095	− 0.270
*BRIEF-P (teachers)*									
Inhibit	53.333	17.162	15	44.114	6.120	35	148.500	0.015(*)	− 0.434
Emotional control	52.400	16.278	15	45.714	3.626	35	207.000	0.236	− 0.211
Shift	48.267	13.572	15	48.853	8.528	34	300.500	0.311	0.178
Working memory	51.533	16.780	15	46.543	7.793	35	222.000	0.392	− 0.154
Planning/organize	51.600	14.744	15	46.686	7.630	35	210.000	0.266	− 0.200
GEC	52.333	18.984	15	44.706	5.761	34	184.500	0.128	− 0.276

*BRIEF-P* Behavioral Rating Inventory of Executive Function, preschool version, *C* Controls; *CGT* Children Gambling Task, *DCCS* Dimensional Change Card Sort test, *GEC* Global

Executive Composite, *HPA* Patients with hyperphenylalaninemia, *M* Mean, *n* Headcount, *r* Rank-biserial correlation, *SD* Standard deviation

* *p* ≤ .01; (*) *p* ≤ .05: values are considered to tend to be significant

### PKU versus MHP

#### Sample characteristics

The demographic and clinical characteristics of the PKU and MHP patients are summarised in Table 4. The two groups are comparable in terms of age, sex, and parental education level. All WPPSI-IV indices (VCI, VSI, FRI, WMI, PSI) and FSIQ are within the expected mean for age (score between 90 and 109) for both groups and there are no significant differences between the two groups. However, IQ scores appear to be lower for most indices in PKU patients than in MHPs (apart from the FRI), with two indices trending towards significance (WMI, moderate effect size, and VSI, small effect size). In terms of metabolic variables, there is a significance in the level of Phe at day 3 (*p* = 0.001) and day 10 of life between the two groups (*p* < 0.001): PKU group has higher Phe level for both indicators. The variability of Phe levels during the lifetime (SD Phe) is also significant: the PKU group showed increased variability compared to the MHP group (*p* < 0.001). A trend toward significance also appears in the mean Phe/Tyr ratio, with the PKU group having a higher ratio than the MHP group (*p* = 0.013). There is no significant difference between the two groups (PKU vs MHP) for the other metabolic variables (mean Phe during life, inclusion Phe and inclusion Phe/Tyr ratio).

**Table 4:** Demographic and clinical data of the MHP and PKU groups

	MHP				PKU				Mann–Whitney			Effet size
	M	SD	Range	*n*	M	SD	Range	*n*	U	*p*		
Age (months)	56.3	9.7	43–71	11	50.4	10.5	36–68	12	87.000	0.206		0.318
Mean PEL (years)	13.2	2.3	8.5–18.5	11	12.5	2.2	10–18	12	79.000	0.440		0.197
Girls/Boys	6/5			11	10/2			12	Chi^2^ = 2.246	0.134		
Treatments												
Hypoprotidic diet	0/11			11	9/12			12				
Amino acid supplement	0/11			11	11/12			12				
Tetrahydrobiopterin	1/11			11	4/12			12				
Normoprotidic diet	11/11			11	0/12			12				
Phe D3 (μmol/l)	210	50	156–312	8	523	243.3	222–960	11	4.500	0.001**		− 0.898
Phe D10 (μmol/l)	242	52	186–348	11	1185	741.8	390–2700	12	0.000	< .001**		− 1.000
Inclusion Phe (μmol/l)	292	170	138–732	11	437	253.6	156–984	12	39.500	0.109		− 0.402
Inclusion Phe/Tyr Ratio	5	3	2–11	11	12	11.6	1–39		37.000	0.079		− 0.439
Mean Phe (μmol/l)	276	78	173–431	11	322	104.4	219–618	12	46.000	0.235		− 0.303
Mean Phe/Tyr Ratio	6	7	2–23	11	13	12.2	2–41	12	37.000	0.079		− 0.439
Variability Phe (μmol/l)	106	106	28- 393	11	171	96.1	66–364	12	29.000	0.023(*)		− 0.561
VCI	110.1	10.8	98–126	11	102	13.8	87–129	12	90.500	0.138		0.371
VSI	101.9	9.4	82–112	11	93.1	10.9	72–106	11	90.000	0.055		0.488
FRI	94.6	10.6	80–111	8	96.7	5.9	89–107	6	21.500	0.795		− 0.104
WMI	100.5	7.3	85–109	11	91.7	10.4	75–109	10	83.000	0.051		0.509
PSI	97.7	13.4	77–118	6	92	7	82–100	6	23.500	0.420		0.306
FSIQ	102.7	10.7	88–118	11	95.2	11.8	77–115	12	92.500	0.109		0.402

*FRI* Fluid reasoning index, *FSIQ* Full-scale intelligence quotient, *Inclusion Phe* Phe level at the study inclusion, *Inclusion Phe/Tyr ratio* Ratio Phe/Tyr at the study inclusion, *M* Mean; *Mean Phe* Mean Phe level during lifespan, *Mean Phe/Tyr ratio* Mean Phe/Tyr level during lifespan, *MHP* Patients with moderate hyperphenylalaninemia, *n* Headcount, *PEL* Parental education level, *Phe D3* Phe level at day 3 of live, *Phe D10* Phe level at day 10 of live, *PKU* Patients with phenylketonuria, *PSI* Processing speed index, *SD* Standard deviation, *Variability Phe* Phe level variability during lifespan, *VCI* Verbal comprehension index, *VSI* Visual spatial index, *WMI* Working memory index

^**^p ≤ .001; *p ≤ .01; (*) *p* ≤ .05: values are considered to tend to be significant

#### Executive functions

The results for the tests and questionnaire for the PKU and MHP patients are presented in Table 5. Differences between the two groups were observed for 3 executive tasks, systematically to the disadvantage of PKU patients: lower backward visuospatial span (*p* = 0.008; moderate effect size), lower forward digit span (*p* = 0.042; small effect size), increased error score on the Sun-Moon Stroop (*p* = 0.010, small effect size). The two groups are comparable in the other outcomes. Regarding the parents’ questionnaires (BRIEF-P), there are no significant differences between the two groups. Regarding the teachers’ questionnaires, the inhibition scale score tended to be higher in patients with MHP (*p* = 0.029, moderate effect size).

**Table 5:** Score on executive performance-based tests in preschool children with MHP and PKU

	MHP			PKU			Mann–Whitney		Effet size
	M	SD	*n*	M	SD	*n*	U	*p*	r
*Sun-moon stroop*									
Interference score	− 0.360	0.222	10	− 0.435	0.241	10	59.000	0.529	0.180
Error score	− 2.200	2.440	10	4.800	8.483	10	16.000	0.010*	− 0.680
*Hand-game*									
Uncorrected errors	2.333	2.449	9	2.250	3.671	12	62.000	0.581	0.148
Self-corrected errors	0.444	1.014	9	1.000	1.758	12	46.500	0.533	-0.139
*Digit span*									
Forward span	3.700	0.675	10	2.909	1.044	11	81.000	0.042(*)	0.473
Backward span	1.500	1.080	10	0.636	1.120	11	76.500	0.099	0.391
*Spatial Span*									
Forward span	3.600	0.699	10	3.083	1.084	12	85.500	0.070	0.425
Backward span	2.200	1.317	10	0.667	0.985	12	98.000	0.008*	0.633
*DCCS*									
Success score	2.400	0.516	10	2.083	0.515	12	77.000	0.182	0.283
*Brixton preschool*									
Total success score	14.500	6.671	10	11.500	6.557	12	77.500	0.260	0.292
*CGT*									
Overall proportion score	0.232	0.378	10	0.253	0.456	12	46.500	0.391	-0.225
*BRIEF-P (parents)*									
Inhibit	57.000	17.049	10	43.875	10.260	8	59.000	0.100	0.475
Emotionnal control	56.200	14.297	10	48.375	7.386	8	51.000	0.349	0.275
Shift	54.500	15.299	10	54.250	15.563	8	42.000	0.894	0.050
Working memory	56.800	15.237	10	47.500	10.529	8	56.000	0.168	0.400
Planning/organize	58.800	16.565	10	46.375	8.684	8	57.000	0.138	0.425
GEC	58.400	17.212	10	46.375	9.855	8	56.500	0.155	0.413
*BRIEF-P (teachers)*									
Inhibit	57.333	17.776	9	47.333	15.718	6	46.000	0.029(*)	0.704
Emotionnal control	54.444	20.397	9	49.333	7.581	6	27.000	1.000	0.000
Shift	49.222	16.843	9	46.833	7.600	6	29.000	0.842	0.074
Working memory	56.000	20.378	9	44.833	5.845	6	38.500	0.192	0.426
Planning/organize	55.333	17.783	9	46.000	6.325	6	38.000	0.207	0.407
GEC	56.556	22.930	9	46.000	9.359	6	40.000	0.140	0.481

*BRIEF-P* Behavioral Rating Inventory of Executive Function, preschool version, *C* Controls, *CGT* Children Gambling Task, *DCCS* Dimensional Change Card Sort test, *GEC* Global Executive Composite, *M* Mean, *MHP* Patients with moderate hyperphenylalaninemia *n* headcount, *PKU* Patients with phenylketonuria, *r* rank-biserial correlation, *SD* Standard deviation

*p ≤ .01; (*) *p* ≤ .05: values are considered to tend to be significant

### Correlation between metabolic variables and executive functions (performance-based tests and BRIEF-P ratings)

In the overall HPA group, there was a negative and significant correlation between the teaching inhibition index in the BRIEF-P questionnaire and the variability of the Phe over time (*r* = − 0.784, *p* < 0.001). In the PKU group, there was a negative and significant correlation between the overall score in Brixton preschool and the Phe level at inclusion (*r* = − 0.749, *p* 0.005). The higher the levels, the lower the preschool Brixton score and the higher the inhibition index. In the MHP group, there was a positive and significant correlation between the overall DCCS score and the average lifetime Phe rate. The higher the average Phe rate, the higher the DCCS success score for children with MHP. The other correlations were not significant.

## Discussion

The objective of this study was to investigate EFs in children with HPA children and the possible impact of certain metabolic variables on their development in accordance with the new classification recommendations between PKU and MHP [1, 2]. Furthermore, the approach used to study executive functions included combining performance-based tests with daily life questionnaires, including perspectives from parents and the school environment.

For the HPA group, the pattern of results in our study does not support our initial hypothesis suggesting early executive difficulties compared to the general population. Rather, the results show some variability in the HPA group depending on the tasks considered. In performance tests, patients manifested difficulties in the Hand-Game test, suggesting possible deficiencies in the inhibition of a motor response: they make more errors (trend) and self-correct significantly less, which may suggest a relative lack of control of the action. In contrast, HPA children perform better in three tests assessing verbal inhibition (Sun-Moon Stroop, error score), cognitive flexibility (DCCS) and affective decision-making (CGT, trend only). Finally, they performed similarly to controls on all other measures (i.e., the majority) of visual and auditory verbal working memory (forward and backward digit span, forward and backward visuospatial span), verbal inhibition (Sun-Moon Stroop interference score), and flexibility (preschool Brixton test). On the daily life questionnaires (BRIEF-P), there was an equivalent level of reports from both parents and teachers, apart from increased difficulties identified by teachers on the inhibition scale. This significant heterogeneity in the results could be explained by the fact that preschool children are likely to show significant variability in their motor and cognitive development, making it impossible to identify a specific profile. Our results differ from the only study that compared preschool MHP and PKU patients [16], which showed group-wide executive impairment in inhibition and working memory. However, the Phe levels considered were higher and did not yet follow the new classification recommendations. In fact, in our sample of children with HPA, mean Phe levels were quite low and according to current management recommendations (< 360 µmol/L: 299.831 + -93.526), a threshold considered likely to prevent cognitive disorders and especially executive disorders [9, 12, 30].

When comparing the results between the two subgroups of patients, our data confirm that there are possible early executive difficulties, but that they vary significantly according to the type of hyperphenylalaninemia (PKU or MHP), which tends to confirm our second hypothesis. In fact, three executive indicators referring to cognitive inhibition, verbal working memory, and visuospatial working memory were significantly worse for PKU children than for MHP’s, on three different tests: the Sun-Moon Stroop error score, the forward digit span, and the backward spatial span. These data support the idea that executive difficulties appear early in PKU children, in contrast to MHP children. Our results appear to be consistent with previous research that has investigated the executive functions of preschool PKU children using performance tests, both in comparison to control subjects and to test norms [12–14, 17]. For MHP children, our results are partly consistent with previous data in preschoolers. Indeed, the study by Sharman et al. [15] found no evidence of executive impairment in their longitudinal study that included 4 patients with MHP (according to the new recommendations). In contrast, Diamond et al. [16] showed a continuum of impairment between PKU and MHP compared to control subjects. However, these outcomes should be interpreted while recognizing that patients with MHP had Phe levels up to 600 µmol/L (new recommendations 120 µmol/L > Phe < 360 µmol/L), and executive impairment mainly observed in children with MHP with the highest levels.

The executive impairment that preferentially affects patients with PKU could be explained by the nature of the enzyme deficiency, which is more severe in these patients. Despite strict diet control, the "toxic metabolites" pass the blood–brain barrier and could affect EFs earlier than in the case of children with MHP (in case of intercurrent diseases such as fever, virus, etc.). In fact, this phenomenon is likely to lead to an alteration in intracerebral protein synthesis and the synthesis of certain neurotransmitters, particularly dopaminergic ones. Dopamine has an important role in the frontal-subcortical circuits underlying the emergence of executive functions [16, 31]. Similarly, excess Phe has a negative impact on myelin production and can lead to white matter lesions, especially in the frontal lobe, in the form of T2 hyperintensity [2, 32]. On the contrary, MHP patients have residual enzymatic activity that would allow them to reduce the metabolic consequences of the deficit and, to maintain lower concentrations of Phe [3]. This would limit the cognitive impact and delay the potential onset of executive difficulties. Analysis of the intellectual profile of our sample provides additional answers to this issue. Indeed, while PKU and MHP children are comparable in all IQ indicators with scores in the normal range, the WMI tends to be significantly lower in patients with PKU (*p* = 0.051, moderate effect size), which could confirm a particular or even specific fragility or EFs, at an early age, in the case of PKU. This working memory impairment is also found in the study by de la Parra et al. [33] when they analysed intellectual efficiency scores in school aged PKU and MHP children. Specifically, the intellectual profile was similar between the two groups of patients despite more pronounced difficulties in PKU children. It is therefore quite possible that executive difficulties are not yet visible in younger children with MHP because of partial enzymatic impairment but that they may appear over time. Two recent reviews of the literature point in this direction and report indicators of executive dysfunction in older MHP patients in terms of working memory and inhibition [34, 35].

However, our results show that poorer executive performance in preschool PKU patients compared to MHP patients only affects 3 of the 7 performance-based tasks and that there are no significant differences in the daily life questionnaires of parents or teachers. Therefore, the executive difficulties identified in some tests are not found in daily life. These data do not compare well with previous work in the literature, as daily life questionnaires have only been used once in preschool children and only those with PKU [14]. In that study, parents' reports were higher compared to controls, but only on the flexibility and control scales, which is not the case in our results. Alternatively, the only study that used a daily life questionnaire in school-aged MHP patients (BRIEF) did not present executive reports from parents [15], in contrast to what has been shown at this age in PKU [14, 36, 37]. In view of these data, it is possible that the differential in executive impairment between PKU and MHP may not yet be sufficiently noticeable in young children by family members and in the school environment, since EFs are still relatively unused in daily life during this period.

Finally, regarding the links between metabolic variables and EFs, the hypothesis is only partially tested. Indeed, significant correlations (*p* ≤ 0.01) sporadically emerged between the inhibition index at the BRIEF-P teacher version and the variability of rates over the lifespan: the greater the variability of rates, the more inhibition complaints there are among teachers of HPA children. More specifically, in the PKU subgroup, a negative and significant correlation was found between Brixton preschool and Phe level on day of inclusion. Therefore, the higher the Phe level, the more difficult PKU subjects will be to engage in flexibility skills on this test. Conversely, in children with MHP, a positive and significant correlation was found between the global score of the DCCS and the average rate of Phe during life. It seems that the higher the average rates, the more efficient the flexibility capacities required by this test. Moreover, no correlation was found between the metabolic variables and the 3 executive tests where PKU patients performed worse than MHP patients. This is partly consistent with data from the literature, since several studies have also shown negative correlations between Phe levels at inclusion and EFs in preschoolers with HPA [13, 16, 17]. Similarly, variability in rates throughout life can impact the extent of executive impairment in PKU [12, 16, 30]. However, there is also evidence that average Phe [13] or average Phe/Tyr ratio appears to correlate with some executive impairment in children with PKU [16, 38]. The sporadic presence of correlations does not rule out their import as a predictor of executive difficulties in HPA. However, it is possible that due to the young age of the patients, these metabolic indicators lack sensitivity. Adherence to treatment is likely to be most optimal in the early years of life before greater variability in levels emerges during school age and adolescence [39].

This study has several limitations that must be considered in the scope of the outcomes. Firstly, the sample size remains small (HPA preschoolers = 23) and limits the statistical power of the analyses carried out. It should be noted, however, that the sample remains large for a rare disease and for the age group investigated (3–5 years). Moreover, our population does not include patients lost to follow-up, with potential diet failures. These children have a metabolic control in the management recommendations, which may be a potential bias in the interpretation of our results in view of our hypothesis: the worse the metabolic control the worse the outcome scores should be. Furthermore, an unaffected sibling’s control group would have been ideal for a better matching of educational and socioeconomic level. However, the socioeconomic level was considered in the constitution of the control group, which limits the potential bias. In addition, the comparison of results between the 3 subgroups (PKU, MHP and controls) would have been interesting but it was not privileged because of the variability of the performances on such small numbers and by the random character of the effects that this type of comparison generates. Finally, the tests administered are mostly experimental tests, due to the lack of clinically validated tools for preschool children. Therefore, these tasks may lack sensitivity. The ideal would be to create a standardized battery for the evaluation of executive functions in preschoolers based on a unitary approach to executive functions at this period [18]. To consider the fatigability of the youngest children, we should propose 3 or 4 tests that have already shown their sensitivity in various clinical situations [40], such as the DCCS test [41], the Sun-Moon Stroop [42] or the Hand-Game test [20].. It should also be borne in mind that executive difficulties generally become more pronounced with age and that the impact of metabolic variables also modulates with age, and more difficult compliance with metabolic control [39]. Therefore, it is crucial to investigate EFs in school-aged patients with MHP and PKU, using performance tests and daily life measures, while measuring potential links with different metabolic indicators. Ideally, longitudinal follow-up is the best option, to gain a developmental perspective of the executive profile of PKU and MHP patients.

## Conclusions

This study confirms the existence of early executive difficulties in PKU patients, which do not seem to affect MHP children. These difficulties are identified through some performance-based tests but are not perceived by parents or teachers at this stage of development (using BRIEF-P questionnaires). The comparable intellectual profile of PKU and MHP children suggests a possible specific early fragility of EFs in PKU and suggests the importance of their clinical identification in early childhood. Finally, although there is no overall link between the executive profile of the patients and the various metabolic variables considered in this study, the sporadic correlations identified for the Phe level at inclusion, the mean Phe level, and the variability of Phe levels throughout life suggest possible links, which remain poorly predictive for this period of life, but which could constitute useful benchmarks for subsequent neurodevelopmental follow-up. For this, longitudinal studies rather than cross-sectional studies of the same cohort of patients at different ages would be necessary. This would make it possible to refine the dynamics of the onset of executive disorders and their possible causes to propose appropriate treatment.

## Data Availability

The data that support the findings of this study are available from the corresponding author upon reasonable request.

## Abbreviations

*CGT*: *Children's Gambling Task*
*DCCS*: *Dimensional Change Card Sort*
EFs: Executive functions
EMI: Emergent Metacognition Index
FI: Flexibility Index
FRI: Fluid Reasoning Index
FSIQ: Full Scale Intelligence Quotient
GEC: Global Executive Composite
HPA: Hyperphenylalaninemia
ICI: Inhibitory Control Index
IQ: Intelligence Quotient
M: Mean
MHP: Moderate hyperphenylalaninemia
PAH: Phenylalanine Hydroxylase
Phe: Phenylalanine
PKU: Phenylketonuria
PSI: Processing Speed Index
SD: Standard Deviation
VCI: Verbal Comprehension Index
VSI: Visual Spatial Index
WMI: Working Memory Index
WPPSI-IV: Wechsler Preschool and Primary Scale of Intelligence, Fourth Edition
